# Food allergy has no negative impact on children’s school performance: A Swedish sibling and co-twin control study

**DOI:** 10.1016/j.jacig.2024.100380

**Published:** 2024-12-04

**Authors:** Cecilia Lundholm, Hanna Karim, Awad I. Smew, Michael Silverman, Tong Gong, Bronwyn K. Brew, Catarina Almqvist

**Affiliations:** a Department of Medical Epidemiology and Biostatistics, Karolinska Institutet, Stockholm, Sweden; b Paediatric Allergy and Pulmonology Unit, Astrid Lindgren Children’s Hospital, Karolinska University Hospital, Stockholm, Sweden; c Department of Perioperative Medicine and Intensive Care, Karolinska University Hospital, Stockholm, Sweden; d Department of Psychiatry, Icahn Medical School at Mount Sinai, New York, NY; e School of Medicine and Public Health, University of Newcastle, Newcastle, Australia

**Keywords:** Academic performance, adolescents, anaphylaxis, children, food allergy

## Abstract

**Background:**

Food allergy has been shown to negatively impact children’s mental health and quality of life. However, its impact on school performance is unknown.

**Objective:**

We aimed to investigate whether food allergy, severe and nonsevere, is associated with school performance when accounting for measured and unmeasured familial factors.

**Methods:**

This was a register-based cohort study, with sibling controls, including all children born in Sweden 2001-5 (n = 456,164) with food allergy information based on hospital visits and prescriptions, grades, and national test results from all Swedish schools and confounders. Primary exposure was food allergy severity (none, nonsevere, or severe) in school years 7-9, and the primary outcome was total grades from year 9, with secondary exposures/outcomes also at younger ages. The primary outcome was analyzed by linear regression and, for sibling/twin control analyses, fixed effect linear regression. Results were replicated in a twin cohort (n = 31,609).

**Results:**

In unadjusted and analyses adjusted for measured confounders, children with severe food allergy appeared to have better total grades than children without food allergy (β_unadjusted_ = 10.6 [95% confidence interval (CI), 8.6, 12.6] and β_adjusted_ = 5.5 [95% CI, 3.7, 7.4]). When also adjusting for unmeasured confounders shared by siblings, the difference was close to null and statistically nonsignificant (β_sibling_ = 1.6 [95% CI, −1.5, 4.7]; for nonsevere food allergy, β_sibling_ = −0.0 [95% CI, −2.2, 2.1]). The twin cohort results were similar.

**Conclusions:**

We found no consistent evidence of a negative effect of food allergy, either severe or nonsevere, on school performance when adjusting for measured and unmeasured confounders shared by siblings.

Food allergy affects up to 10% of the population and may in severe cases cause anaphylaxis.^1^, ^2^, ^3^ Research has indicated that the childhood prevalence of food-induced anaphylaxis is 0.25% to 1.2%.^4^ Living with food allergy may be burdensome for children and adolescents.^5^ It has been shown they have lower health-related quality of life^6^^,^^7^ as well as more internalizing problems,^8^ anxiety,^9^ and depression^10^ than their peers. Further, there is evidence of an association between internalizing disorders in children and cognitive performance^11^ or school performance.^12^ It is therefore possible that food allergies could affect school performance, in particular severe food allergy with risk of anaphylaxis. Several studies have investigated the potential effects of other atopic diseases on school performance,^13^, ^14^, ^15^, ^16^, ^17^, ^18^, ^19^ but to our knowledge, only one study investigated the association with food allergy,^20^ and that study did not differentiate between severe food allergy and milder variants.

Research on food allergy may be challenging regarding how to measure the disease. The prevalence of self-reported or parent-reported food allergy is usually much higher than food allergy based on provocation tests, skin prick tests, or food allergy symptoms in combination with IgE response.^1^, ^2^, ^3^ Research on severe food allergy is further complicated by its rarity, thus requiring large study populations. A solution to this could be to use national health register data reported from hospital-based specialist care with food allergy diagnosed by specialists, which is generally based on either provocation, skin prick, or IgE test.^21^ Moreover, children who have had severe allergic reactions are unlikely to be missed by the health care system because they require medical treatment, with that information captured in the register.

We aimed to investigate if children with food allergy have lower school performance than children without food allergy in 2 cohorts: first, a population-based national cohort using data from hospital-based specialist care and prescribed medication, and second, a twin cohort with questionnaire-based information on food allergy, both in combination with subject-level registry data on school performance. Considering that factors influencing parental health–seeking behavior and reporting of food allergies may also influence their children’s school performance, it is important to account for such familial factors. This can be achieved by using family-based study designs, where exposed individuals (those with food allergy) are compared to their unexposed family members (those without food allergy) regarding the outcome. Such designs account for confounding factors that siblings/twins share, including parental behavioral and genetic factors.^22^ We aimed to account for familial factors by comparing individuals with food allergy to both their unexposed full siblings and their co-twins.

## Methods

### Study design and study populations

This cohort study included 2 study populations, one based on the Swedish national registers only and the other a twin population from the Swedish Twin Registry,^23^ with questionnaire data combined with register data for replication of results. All register data were linked by Swedish identity number and are therefore unambiguous.^24^ Informed consent was obtained from the parents of the twins was but was waived by the Swedish ethical review authority, which approved the study.

### Register-based cohort

In the register-based cohort, we included all children born in Sweden during 2001-5 according to the Total Population Register (RTP; N = 495,085),^25^ including information on birth date, death date, sex, immigration background, and parents’ identity. The cohort was linked to the Migration Register with dates of emigration and immigration, the school year 9 register with information on grades in year 9 and eligibility to upper secondary school (USS), the registers for national test results in school years 3, 6, and 9, the longitudinal integrated database for health insurance and labor market studies (LISA)^26^ with information on highest attained education and income, the National Patient Register (NPR)^27^ with information on specialist care visits (coverage, 100% inpatient visits from 1987 and ∼80% outpatient visits from 2001), including visit dates and diagnoses according to the International Classification of Diseases, Tenth Revision (ICD-10), and the Swedish Prescribed Drug Register (SPDR) with information on all prescribed drugs (by Anatomical Therapeutic Chemical [ATC] code) dispensed at pharmacies in Sweden since July 1, 2005.

We excluded children with missing identity of both parents (n = 160), who died (n = 2,311), or who emigrated (n = 25,448) before June 30 of the year they would have graduated from compulsory school (year 9), and children with a diagnosis of developmental delay or chromosomal aberration that is likely to affect school performance (ICD-10: F70-F89, D82.1, Q87.1, Q87.8, Q90-Q93, Q98-99; n = 7,614). Further, we excluded children missing information on all outcome measures (n = 2,456). The final register-based study population consisted of 456,164 children.

### Twin cohort

The twin cohort was based on the Childhood and Adolescent Twin Study in Sweden (CATSS), which is an ongoing study.^28^ In CATSS, all twins born in Sweden since July 1992 have been invited to participate in the study at 9 years of age (in the beginning, 12-year-olds were also invited). The parents are asked to respond to questionnaires including questions about both somatic and psychiatric health, including allergies. Twin pairs are excluded if one or both twins die or emigrate before being invited to the study, or if the parents are unable to answer the questionnaire (telephone interview) as a result of disability or not being fluent in Swedish. Twin pairs are also excluded if one of them has a severe disability that makes the questions inapplicable.

For this twin cohort, we included all twins from CATSS interviewed until May 2020, which provided a cohort of 34,372 twins born 1992-2010. Twin pairs were excluded on the same grounds as the register-based cohort (14 died, 469 emigrated, and 489 had developmental delay/chromosomal aberration). Further, we excluded twins without answers to the main questions about food allergy (n = 58) and twins not having information on any of the outcome measures by year 2020 (n = 1,733). Co-twins not fulfilling the exclusion criteria were kept in the cohort. The final twin population included 31,609 subjects. Twins with the primary outcome grade point sum in year 9 were born 1992-2006. Those with the secondary outcome national test results in year 9 were born 1992-2004, in year 6 1999-2006, and in year 3 1999-2010.

### Food allergy

Our primary exposure measurement was food allergy severity in school years 7-9. The variable was defined in slightly different ways in the 2 cohorts.

In the register-based cohort, severity was defined on the basis of a diagnosis of food allergy (ICD-10: Z91.0A, Z91.0B, Z91.0C, Z91.0D, Z91.0E) or anaphylaxis due to food allergy (ICD-10: T78.0) in NPR in combination with dispensed prescriptions of adrenaline autoinjectors (ATC: C01CA24) in SPDR, with the following categories:

*No food allergy*—No diagnoses of food allergy or anaphylaxis due to food allergy ever before July 1 of the year the child graduated from school year 9.
*Nonsevere food allergy*—At least one specialist care visit ever with a food allergy diagnosis before July 1 of the year the child graduated from school year 9, but no diagnosis of anaphylaxis and no dispensed prescription of adrenaline autoinjector during the 3 years before that date.
*Severe food allergy*—At least one specialist care visit ever with a food allergy diagnosis before July 1 of the year the child graduated from school year 9, in combination with at least one diagnosis of anaphylaxis and/or one dispensed prescription of adrenaline autoinjector in SPDR during the 3 years before that date.

Secondary exposure variables are provided in the Online Repository at www.jaci-global.org.

In the twin cohort, parents were asked if the child had a food allergy other than gluten or lactose intolerance, and if so, whether the allergy had been diagnosed by a doctor at 9 or 12 years of age, depending on age at interview. The primary exposure variable—food allergy severity in years 7-9—was categorized as follows in the twins:

*No food allergy*—No parent-reported food allergy at 9 years of age.
*Nonsevere food allergy without doctor’s diagnosis*—Parent-reported food allergy but no doctor’s diagnosis by age 9 or 12 years.
*Nonsevere food allergy with doctor’s diagnosis*—Parent-reported food allergy that, according to the parent, had also been diagnosed by a doctor by 9 or 12 years.
*Severe food allergy*—Parent-reported food allergy by 9 or 12 years of age, in combination with at least one diagnosis of anaphylaxis in the NPR and/or one dispensed prescription of adrenaline autoinjectors in SPDR during the 3 years before July 1 of the year the child graduated from school year 9.

Corresponding food allergy exposure variables were retrieved for school years 3 and 6 on the basis of additional information from the NPR and SPDR, to be used for outcomes in years 3 and 6, respectively for both cohorts. Details and results for secondary exposure variables (food allergy ever by school year 9, binary and severity) can be found in the Online Repository at www.jaci-global.org.

### Measures of school performance

In Sweden, all schools are free of charge, and the children start school in August the year they turn 7. Consequently, children start year 3 the year they turn 9, year 6 the year they turn 12, and year 9 the year they turn 15. The first 9 years of schooling are compulsory.^29^ After this, the students can apply to USS on the basis of their grades from year 9, if eligible. Admission to popular USS educational programs and schools depends on the grades from year 9. National tests have been run yearly in school year 3 since 2008, year 6 since 2011, and year 9 since 1997 to ensure comparable evaluation and grading of students throughout the country. All Swedish schools must report grades and national test results to Statistics Sweden.

We used the same outcome measures in both the register-based and twin cohorts. Our primary outcome measure was grade point sum from year 9. Grade point sum is a sum of grades in 16 subjects, based on a numeric transformation from the original ordinal grades ranging from F = 0 to A = 20. Grade point sum has a range of 0 to 320 points.

Secondary outcomes were noneligibility to USS, which happens if failing (grade F) in any of the 3 core subjects (Swedish, mathematics, English) in year 9, as well as results from national tests in years 3 (Swedish and mathematics), 6 (Swedish, mathematics, English) and 9 (Swedish, mathematics, English). In year 3, the national test results are coded as passing all modules in the subject versus failing at least one module. In years 6 and 9, the result from the tests are grades transformed into discrete numeric variables in the same way as for the grade point sum, resulting in a range of 0 to 20.

### Covariates

Covariates were the same in both the register-based and twin cohorts and were selected on the basis of the literature on risk factors for food allergy,^30^ subject matter knowledge about factors influencing health care–seeking behavior, and school results by using a directed acyclic graph (see Fig E1 in the Online Repository at www.jaci-global.org). The selected covariates were sex (from RTP), birth year (from RTP), mother’s and father’s education (from LISA), family income (from LISA, log transformed, continuous), and immigrant parents (from RTP). Categorical covariates were categorized as shown in Table I.

**Table I:** Background characteristics by food allergy severity in years 7-9

Characteristic	Register-based cohort						Twin cohort							
	No disease		Nonsevere		Severe		No disease		Nonsevere, without doctor’s diagnosis		Nonsevere, with doctor’s diagnosis		Severe	
	No.	%	No.	%	No.	%	No.	%	No.	%	No.	%	No.	%
	443454	97.1	9548	2.1	3558	0.8	28656	90.7	1184	3.7	1411	4.5	194	0.6
Child’s sex														
Boy	226000	51.0	5165	54.1	2014	56.6	233179	51.1	543	45.9	783	55.5	100	51.5
Girl	217058	49.0	4383	45.9	1544	43.4	222985	48.9	641	54.1	628	44.5	94	48.5
Immigrant parents														
Both born in Sweden	341446	77.1	7200	75.4	2760	77.6	24183	84.4	1016	85.8	1215	86.1	168	86.6
One parent immigrant	52633	11.9	1232	12.9	488	13.7	2889	10.1	121	10.2	132	9.4	21	10.8
Both parents immigrants	48979	11.1	1116	11.7	310	8.7	1584	5.5	47	4.0	64	4.5	5	2.6
Mother’s education														
Lower secondary school <9 years	9869	2.2	191	2.0	32	0.9	180	0.6	8	0.7	8	0.6	2	1.0
Lower secondary school 9 years	30530	6.9	604	6.3	164	4.6	1538	5.4	56	4.7	79	5.6	7	3.6
USS 1-2 years	76340	17.2	1518	15.9	528	14.8	6688	23.3	259	21.9	277	19.6	38	19.6
USS 3 years	118395	26.7	2505	26.2	896	25.2	6192	21.6	245	20.7	279	19.8	35	18.0
College/university <3 years	65224	14.7	1500	15.7	558	15.7	5310	18.5	223	18.8	285	20.2	43	22.2
College/university ≥3years	134915	30.5	3085	32.3	1305	36.7	8343	29.1	372	31.4	462	32.7	68	35.1
Postgraduate education	4849	1.1	102	1.1	62	1.7	300	1.0	18	1.5	20	1.4	1	0.5
Missing	2936	0.7	43	0.5	13	0.4	105	0.4	3	0.3	1	0.1	0	0.0
Father’s education														
Lower secondary school <9 years	8131	1.8	160	1.7	41	1.2	331	1.2	19	1.6	14	1.0	5	2.6
Lower secondary school 9 years	41613	9.4	826	8.7	252	7.1	2567	9.0	113	9.5	112	7.9	17	8.8
USS 1-2 years	126589	28.6	2607	27.3	894	25.1	9135	31.9	349	29.5	417	29.6	54	27.8
USS 3 years	96731	21.8	2114	22.1	694	19.5	4971	17.3	203	17.1	280	19.8	28	14.4
College/university <3 years	65362	14.8	1426	14.9	621	17.5	4905	17.1	199	16.8	221	15.7	38	19.6
College/university ≥3years	88592	20.0	2048	21.4	911	25.6	5797	20.2	258	21.8	305	21.6	43	22.2
Postgraduate education	7841	1.8	167	1.7	90	2.5	563	2.0	17	1.4	27	1.9	4	2.1
Missing	8199	1.9	200	2.1	55	1.5	387	1.4	26	2.2	35	2.5	5	2.6

### Statistical methods

We used linear regression to estimate the association between food allergy (all exposure variables) and grade point sum and national test results in years 6 and 9, with robust standard errors to handle model misspecifications. Logistic regression was used to estimate the associations between food allergy exposure and the outcomes noneligibility to USS (modeling odds of being noneligible) and national test results in year 3 (modeling odds of passing all modules). All associations were estimated unadjusted and adjusted for sex, birth year, both parents’ educational levels, family income, and immigrant parents. Further, because family environment and genetics are known to affect school performance, we also used sibling control design (co-twin control in the twin cohort) to estimate the associations accounting for unmeasured familial factors that are shared between siblings. In the twin cohort, we used cluster robust standard errors to account for correlated observations within twin pairs. For the sibling control analyses, we identified all sibling groups (same biological mother and father) within the register-based cohort. The sibling/co-twin control data were analyzed by fixed effects linear regression with robust standard errors (grade point sum and national test results in years 6 and 9) and conditional logistic regression (noneligibility to USS and national test results in year 3).

In the register-based cohort, we also estimated potential effect modification by sex and parental education by including interaction terms with sex and parental education, respectively, parametrized to get group-specific estimates of the associations. Parental educational groups were combined for this purpose (middle school ≤9 years as middle school, USS 1-3 years as USS, and college/university ≥1 year as university). Further, we investigated associations by number of food allergies and types of food.

## Results

For the primary exposure (food allergy in school years 7-9) in the register-based cohort, we found that 2.1% of children had nonsevere food allergy and 0.8% had severe food allergy. In the twin cohort, parents reported that 8.2% of the children had nonsevere food allergy (3.7% without a doctor’s diagnosis and 4.5% with a doctor’s diagnosis), and 0.6% reported severe food allergy. Diagnosed food allergy (nonsevere food allergy in register-based cohort and nonsevere food allergy with doctor’s diagnosis in twin cohort) and severe food allergy were more common among boys than girls, and among children with parents with higher educational level (Table I). In the register-based cohort, mean [standard deviation (SD)] grade point sum was 228 [65] both in the group without food allergy and in the group with nonsevere food allergy, while it was 238 [61] in the severe food allergy group (Table II), with corresponding numbers for the twin cohort (see Table E1 in the Online Repository at www.jaci-global.org).

**Table II:** Descriptive statistics for outcome variables by food allergy severity in register-based cohort

Characteristic	No	Nonsevere	Severe
Food allergy severity in years 7-9			
Grade point sum in year 9, mean [SD]	228 [65]	228 [65]	238 [61]
Noneligibility to USS, no. (%)	40,871 (9.4)	881 (9.2)	223 (6.3)
National test score Swedish year 9, mean [SD]	13.7 [4.1]	13.8 [4.1]	14.3 [3.9]
National test score mathematics year 9, mean [SD]	11.2 [5.6]	11.3 [5.5]	12.1 [5.3]
National test score English year 9, mean [SD]	15.6 [3.8]	15.7 [3.8]	16.2 [3.5]
Food allergy severity in years 1-3			
Passing national test Swedish year 3, passing all modules, no. (%)	332,391 (76.9)	5,341 (76.4)	1,901 (80.7)
Passing national test mathematics year 3, passing all modules, no. (%)	306,487 (70.8)	4,959 (70.7)	1,782 (75.8)
Food allergy severity in years 4-6			
National test score Swedish year 6, mean [SD]	13.3 [4.2]	13.3 [4.1]	13.8 [3.9]
National test score mathematics year 6, mean [SD]	12.8 [5.0]	12.9 [4.9]	13.6 [4.6]
National test score English year 6, mean [SD]	15.0 [4.6]	15.1 [4.6]	15.8 [4.2]

### Grade point sum in year 9 and noneligibility to USS

For the primary exposure food allergy in school years 7-9, unadjusted analyses and analyses adjusted for measured confounders indicated that children with severe food allergy had higher grades (grade point sum) than children without food allergy (unadjusted mean difference = 10.6 [95% confidence interval (CI), 8.6, 12.6], and adjusted mean difference = 5.5 [95% CI, 3.7, 7.4]) (Table III). The difference, however, was smaller and statistically nonsignificant when also adjusting for unmeasured confounders shared within the sibling groups (β = 1.6 [95% CI, −1.5, 4.7]). For noneligibility to USS, the estimates were attenuated when adjusting for measured confounders and changed direction in sibling control analyses, although not statistically significantly (Table III). Similar results were seen for secondary exposures (see Table E2 in the Online Repository at www.jaci-global.org) and in the twin cohort (see Table E3 in the Online Repository). Results for associations by number of food allergies and specific food types are presented in Table E8, also in the Online Repository.

**Table III:** Results from register-based cohort

Grade point sum	Unadjusted			Adjusted∗			Sibling†		
	N	β	95% CI	N	β	95% CI	N	β	95% CI
Food allergy in years 7-9									
No	437,331	0		426,902	0		4,214	0	
Nonsevere	9,548	0.8	−0.5, 2.1	9,310	0.3	−0.9, 1.5	3,001	−0.0	−2.2, 2.1
Severe	3,558	10.6	8.6, 12.6	3,491	5.5	3.7, 7.4	1,196	1.6	−1.5, 4.7

Noneligibility to USS	n/N	OR	95% CI	n/N	OR	95% CI	n/N	OR	95% CI
Food allergy in years 7-9									
No	40,871/436,836	1		39,030/426,466	1		192/400	1	
Nonsevere	881/9,532	0.99	0.92, 1.06	837/9,294	1.02	0.95,1.10	143/284	1.03	0.82, 1.31
Severe	223/3,549	0.65	0.57, 0.74	212/3,482	0.77	0.67,0.89	51/97	1.14	0.77, 1.69

Shown are associations between food allergy and grades (grade point sum) or noneligibility to USS; data are unadjusted, adjusted for measured confounders, and additionally adjusted for confounders shared between siblings. *OR,* Odds ratio.

∗ Adjusted for sex, birth year, mother’s and father’s education, family income and parents’ immigration status.

† Adjusted for sex, birth year and family income; adjusted for familial factors shared by siblings by fixed effects linear regression or conditional logistic regression.

### Results of national tests in years 3, 6, and 9

**Fig 1:**
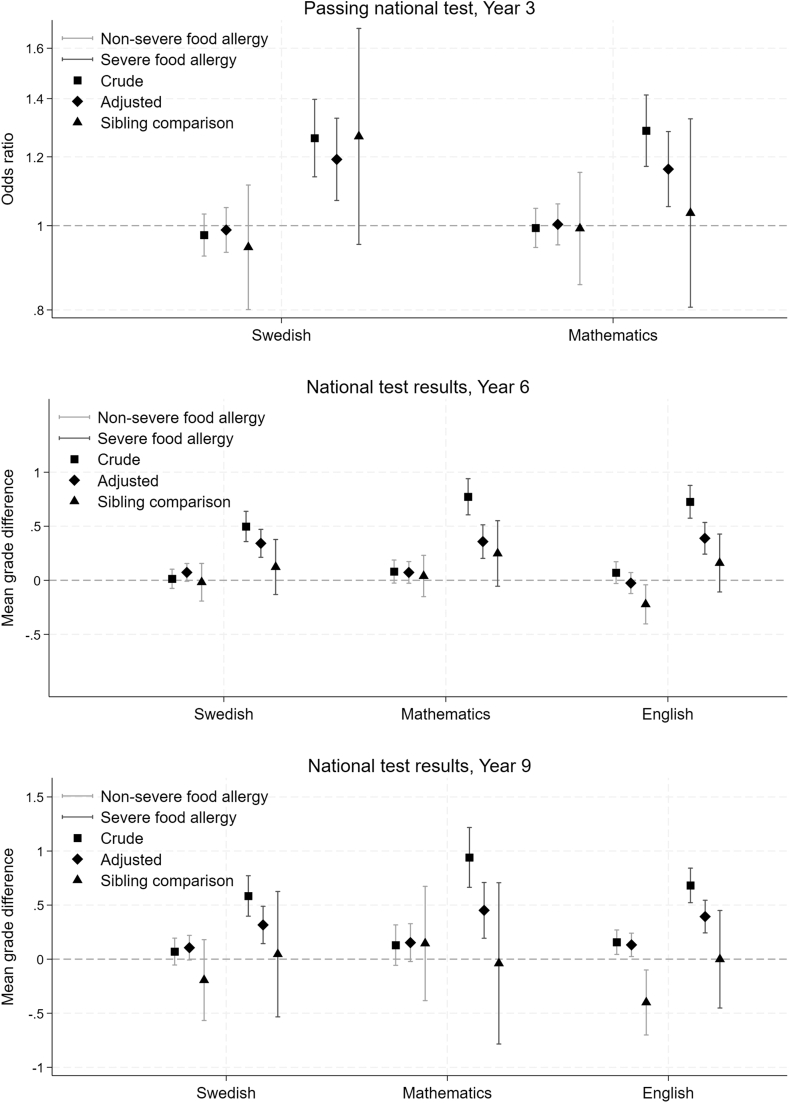
Associations between food allergy and national test results, estimated by logistic regression/conditional logistic regression and linear regression/fixed linear regression, with no food allergy as reference category for exposure. Error bars indicate 95% CIs.

A similar pattern was seen for national test results in the register-based cohort (Fig 1, and see Table E4 in the Online Repository at www.jaci-global.org). The severe food allergy category of primary exposure food allergy severity in school years 7-9 had higher mean test results in all 3 subjects (Swedish, mathematics and English)— for example in Swedish 0.6 points higher mean in the severe food allergy group in unadjusted analyses (95% CI, 0.4, 0.8) and 0.3 points higher in adjusted analyses (95% CI, 0.1, 0.5), which was attenuated to 0.0 points’ difference when adjusting for unmeasured confounders shared within sibling groups (95% CI, −0.5, 0.6). Notably, the results for national tests in English were reversed and statistically significant in the sibling–control analyses for the nonsevere food allergy group in both years 6 and 9: linear regression coefficient (β) = −0.4 (95% CI, −0.7, −0.1) for food allergy severity in school years 7-9 and national test in English in year 9. Similar results were found for secondary exposures (Table E4) and the twin cohort (see Table E5 in the Online Repository).

### Effect modification by sex and parental education

Most analyses indicated no effect modification by sex or parental education, with few exceptions (see Tables E6 and E7 in the Online Repository at www.jaci-global.org). For the primary outcome, the estimated difference between boys with severe food allergy and boys without food allergy was β = 1.3 (95% CI, −2.8, 5.5) and in girls β = 2.5 (95% CI, −2.2, 7.1) (*P* = .47 for sex difference in β) (Table E6). Among children with parents with a middle-school education, only those with severe food allergy (n = 13) had 42.4 points lower grade point sum (β = −42.4 [95% CI, −77.0, −7.9]) compared to children without food allergy, while the corresponding difference for children of parents with university education was in the opposite direction (β = 2.1 [95% CI, −1.3, 5.5], *P* = .009 for difference in β by parental education) (Table E7).

## Discussion

In this large population-based cohort study with sibling controls, we found no consistent associations between food allergy and school performance. Although significant associations were found in the cohort analyses adjusted for important measured confounders, indicating better school performance in children with food allergy compared to those without, the results did not hold when also accounting for unmeasured familial factors in the sibling control analyses. The only significant result in sibling control analyses was for a secondary outcome (national test in English in year 9) and indicated a very small difference, which was not replicated in the twin cohort. Otherwise, when replicating the results in our twin cohort, similar results were found. We also found some statistically significant interactions with parental education, but not sex, although with no consistent pattern.

To our knowledge, the only study on food allergy that was based on a subcohort of our twin cohort, but without differentiating between nonsevere and severe allergy, found results similar to ours—that is, no difference in school performance between children with and without food allergy.^20^ Studies on other atopic diseases, atopic eczema, asthma, and rhinitis have indicated that children and adolescents with these diseases may perform as well or better than their peers without those diseases,^13^^,^^15^, ^16^, ^17^, ^18^ but worse if they have severe disease^14^, ^15^, ^16^^,^^18^ or have symptoms while taking exams.^19^ The results from those studies for nonsevere eczema, asthma, and rhinitis were similar to ours for nonsevere food allergy but were different regarding children with severe disease. In line with our results, the authors of a literature review concluded that although children with chronic diseases are more absent from school, they do not seem to have lower achievement,^31^ while a more recent review concluded that such associations differ depending on illness, severity, and treatment.^32^ The difference between our results and those for severe eczema, asthma, and rhinitis may be explained by children with food allergy, successfully avoiding the allergens that causes reactions, do not need to take medication and may have as good somatic health as their peers without food allergy. For the other atopic diseases, it may be more difficult to avoid the triggers of the disease and may therefore have worse somatic health or need medication that may have side effects. An alternative explanation could be that we, unlike most others, could account for familial factors, including parental factors that may affect both their health-seeking behavior and the school performance of their children. In our previous study on school performance of children with asthma, we also observed a clear attenuation of estimates when accounting for familial factors in sibling comparisons.^13^ The attenuation in estimates could be due to parental factors associated with both health-seeking behavior and school results, including abilities and attitudes toward both health and schooling.

The most important limitation of this study concerns the measurement of food allergy. The registers only include food allergy diagnoses from specialist care in combination with adrenaline autoinjector dispenses. Consequently, although specificity is likely to be high, we misclassify children diagnosed in primary care or not diagnosed at all as not having food allergy, resulting in low sensitivity. In contrast, we may have had high sensitivity but low specificity in the twin cohort, where food allergy exposure was based on parental reports in questionnaire. Considering that, according to Swedish treatment guidelines,^33^ adrenaline autoinjectors are prescribed only for patients with a previous anaphylactic reaction, the extent of misclassification should be limited for severe food allergy in both cohorts. Moreover, we cannot be sure the children will still have their allergies when they start school—again, this is not a problem for severe allergy in years 7-9, as this requires either dispensing an adrenaline autoinjector or a hospital diagnosis of anaphylaxis in those years, although there is a small risk of their being prescribed adrenaline for allergies other than food allergy. Because we only had information on school performance every third year, we cannot exclude temporary negative effects of food allergy on school performance in connection to the onset of food allergy. We cannot completely rule out so-called carry-over effects,^34^ such that the food allergy in one sibling may affect all siblings toward being more responsible and thereby enhanced school performance. Such carry-over effects would lead to conservative estimates in the sibling/twin control analyses.

Important strengths of this study include the sibling/co-twin control analyses that enable us to account for confounding from unmeasured and even unknown confounders shared between siblings, including parental factors affecting reporting in questionnaires and health-seeking behavior. The results from those analyses show that such confounding may be substantial. We also had 2 large cohorts, and in both, data were prospectively recorded. Further, although these cohorts suffer from measurement error in the exposure, similar results were observed irrespective of whether the sensitivity or specificity of the exposure measurement is low.

In conclusion, we did not find evidence indicating that pediatric food allergy negatively impacts school performance. In unadjusted and analyses adjusted for measured confounders, we observed an association between food allergy and improved school performance, which was attenuated and statistically nonsignificant when also accounting for unmeasured familial factors by using siblings and co-twins as comparisons. This highlights the importance of utilizing methods that have already been developed to address the limitations of traditional observational studies.

## Disclosure statement

The Swedish Twin Registry is managed by 10.13039/501100004047Karolinska Institutet and receives funding through the 10.13039/501100004359Swedish Research Council under grant 2017-00641. Financial support was provided from the 10.13039/501100004359Swedish Research Council (grants 2018-02640 and 2023-02327), the Strategic Research Program in Epidemiology at 10.13039/501100004047Karolinska Institutet, the Swedish Heart–Lung Foundation (grants 20180512 and 20210416), the 10.13039/501100010234Swedish Asthma and Allergy Association Research Fund (grant 2020-0008), and the Foundation Frimurare Barnhuset in Stockholm.

Disclosure of potential conflict of interest: The authors declare that they have no relevant conflicts of interest.
