# Supplementary material for: Food allergy has no negative impact on children’s school performance: A Swedish sibling and co-twin control study
Source: J Allergy Clin Immunol Glob. 2024 Dec 4;4(1):100380. doi: 10.1016/j.jacig.2024.100380 (PMC11750558; doi:10.1016/j.jacig.2024.100380)
Supplement: Supplementary Figs and Tables [file mmc1.pdf]

Supplemental material to:

Title: Food allergy does not impact on children's school performance – a Swedish sibling and co-twin control study

Running title: Food allergy does not impact on children's school performance

Authors:

Cecilia Lundholm[1]; Hanna Karim[1,2]; Awad Smew[1]; Michael Silverman[3]; Tong Gong[1]; Bronwyn K Brew[1,4]; Catarina Almqvist[1,2]

Authors Affiliations

[1] Department of Medical Epidemiology and Biostatistics, Karolinska Institutet, Box 281, 171 77 Stockholm, Sweden

[2] Paediatric Allergy and Pulmonology Unit at Astrid Lindgren Children's Hospital, Karolinska University Hospital, C8:28 Eugeniavägen 23, Stockholm, Sweden

[3] Dept of Psychiatry, Box 1230 Icahn Medical School at Mount Sinai, New York, NY 10029 USA

[4] School of Medicine and Public Health, University of Newcastle, Newcastle, NSW, Australia

Contents

Secondary exposure variables 3
Register-based cohort 3
Twin cohort 3
Supplemental figure 5
Supplemental tables 6

Secondary exposure variables

Register-based cohort

In the register-based cohort our secondary exposure measurements were *Food allergy severity ever by Year 9* and *Food allergy ever by Year 9*.

As the primary exposure variable, the secondary exposure variables were based on diagnosis of food allergy (ICD-10: Z91.0A, Z91.0B, Z91.0C, Z91.0D, Z91.0E), anaphylaxis due to food allergy diagnosis (ICD-10: T78.0) and dispensed prescriptions of adrenaline autoinjectors (ATC: C01CA24) in the National Patient Register.

Food allergy severity ever by Year 9 differs from the primary exposure variable in the definition of non-severe and severe food allergy. While the primary exposure variable is aimed to capture the current severity in the last three years prior to the outcome, this secondary exposure variable is intended to capture the highest grade of severity ever by the time of the outcome measures. *Food allergy severity ever by Year 9* had the following categories:

> *No food allergy*: No diagnoses of food allergy or anaphylaxis due to food allergy *ever* before July 1st the year the child graduate from school year 9
>
> *Non-severe food allergy*: At least one specialist care visit ever with a food allergy diagnosis before July 1st of the year the child graduated from school year 9, but no diagnosis of anaphylaxis ever and no dispensed prescription of adrenaline autoinjector ever before that date.
>
> *Severe food allergy*: At least one specialist care visit with a food allergy diagnosis before July 1st of the year the child graduated from school year 9, in combination with at least one diagnosis of anaphylaxis ever and/or one dispensed prescription of adrenaline autoinjector ever in the SPDR before that date.

Food allergy ever by Year 9 was a dichotomous variable:

No: No specialist care visit with a food allergy diagnosis before July 1st of the year the child graduated from school year 9.

Yes: At least one specialist care visit with a food allergy diagnosis ever before July 1st of the year the child graduated from school year 9.

Number of foods causing allergic reaction was defined as the number of different ICD-10 codes among Z91.0A (cow's milk), Z91.0B (egg), Z91.0C (tree nuts/peanuts), Z91.0D (fish/crustacean) and Z91.0E (other food) an individual had, which gave a quantitative variable with the range 0-5.

Allergy to certain food types were defined based on having the different ICD-10 codes Z91.0A-E (yes/no).

Twin cohort

Food allergy severity ever by age school year 9 was categorized as follows in the twins:

> *No food allergy*: No parent-reported food allergy at 9 years of age
>
> *Non-severe food allergy without doctor's diagnosis*: Parent-reported food allergy but no doctor's diagnosis by age 9/12 years.
>
> *Non-severe food allergy with doctor's diagnosis*: Parent-reported food allergy which, according to the parent, had also been diagnosed by a doctor by 9/12 years.

Severe food allergy: Parent-reported food allergy by age 9/12 years, in combination with at least one diagnosis of anaphylaxis ever and/or one dispensed prescription of adrenaline autoinjectors ever in the SPDR before July 1st of the year the child graduated from school year 9.

Food allergy ever at age 9 years (Yes/No) was another secondary exposure variable in the twin cohort, defined as:

No: No parent-reported food allergy (other than lactose intolerance or celiac disease) or allergic reactions to food items by age 9 years.

Yes: Parent-reported food allergy (other than lactose intolerance or celiac disease) or allergic reactions to food items by age 9 years.

Supplemental figure

Figure E1. Directed Acyclic Graph depicturing the research question and associations with related variables.

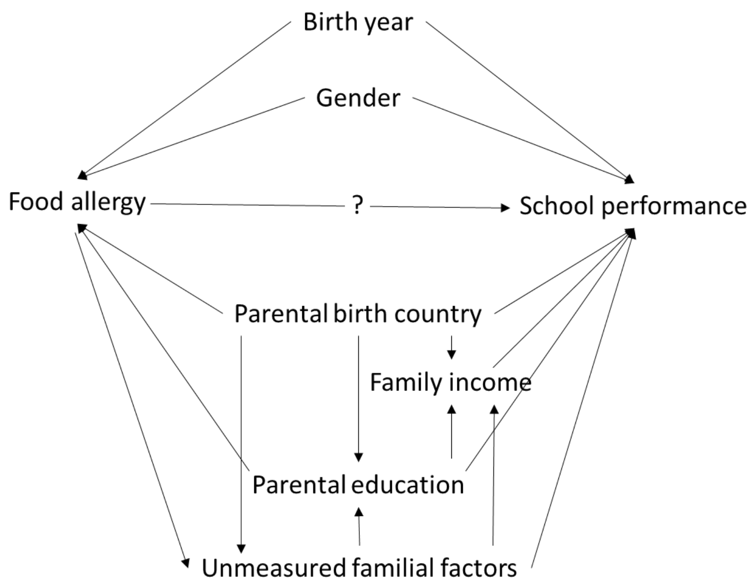

Supplemental tables

Table E1. Results from the twin cohort: Descriptive statistics for the primary school performance variable, Grade point sum and all secondary outcome measures by food allergy severity.

	**Food allergy severity in Years 7-9**			
	**No**	**Non-severe without diagnosis**	**Non-severe with diagnosis**	**Severe**
Grade point sum, mean (SD)	228 (59)	230 (62)	229 (62)	250(50)
Non-eligibility to USS, n (%)	1,683 (7.4)	54 (5.9)	74 (7.1)	10 (5.2)
National test score Swedish Year 9, mean (SD)	13.4 (4.0)	13.7 (3.8)	13.4 (3.9)	14.3 (4.0)
National test score mathematics Year 9, mean (SD)	11.5 (5.5)	11.8 (5.5)	12.1 (5.0)	12.4 (5.8)
National test score English Year 9, mean (SD)	14.8 (4.1)	15.1 (3.9)	14.9 (4.2)	15.8 (3.5)
	**Food allergy severity in Years 1-3**			
	**No**	**Non-severe without diagnosis**	**Non-severe with diagnosis**	**Severe**
Passing national test Swedish Year 3, passing all modules, n (%)	11,739 (78.1)	475 (79.7)	640 (80.1)	56 (88.9)
Passing national test mathematics Year 3, passing all modules, n (%)	10,673 (70.9)	419 (69.9)	565 (71.1)	42 (66.7)
	**Food allergy severity in Years 4-6**			
	**No**	**Non-severe without diagnosis**	**Non-severe with diagnosis**	**Severe**
National test score Swedish Year 6, mean (SD)	13.4 (3.9)	13.6 (3.9)	13.7 (4.0)	14.7 (3.1)
National test score mathematics Year 6, mean (SD)	13.3 (4.9)	13.0 (5.1)	13.5 (4.9)	14.1 (4.5)
National test score English Year 6, mean (SD)	14.8 (4.7)	14.9 (4.9)	15.1 (4.3)	16.0 (4.3)

Table E2. Results from register-based cohort: Associations between food allergy (both primary and secondary exposures) and the primary outcome Grade point sum in Year 9 and the secondary outcome non-eligibility to upper secondary school (USS); unadjusted, adjusted for measured confounders and additionally adjusted for confounders shared between siblings.

		Crude			Adjusted†			Sibling‡	
**Grade point sum in Year 9**		**β**	**95% CI**		**β**	**95% CI**		**β**	**95% CI**
Food allergy in Years 7-9:									
No	22,682	0.0	[0.0,0.0]	22,364	0.0	[0.0,0.0]	1,321	0.0	[0.0,0.0]
Non-severe without doctor's diagnosis	919	2.2	[-1.9,6.3]	892	1.2	[-2.5,4.9]	677	0.8	[-2.7,4.4]
Non-severe with doctor's diagnosis	1,048	1.7	[-2.1,5.5]	1,027	1.6	[-1.8,5.1]	667	-0.3	[-3.6,3.1]
Severe	194	22.0	[15.0,29.0]	189	18.9	[12.9,24.9]	131	-0.3	[-8.4,7.9]
Food allergy by Year 9:									
No	22,682	0.0	[0.0,0.0]	22,364	0.0	[0.0,0.0]	1,342	0.0	[0.0,0.0]
Non-severe without doctor's diagnosis	916	2.0	[-2.1,6.1]	889	1.1	[-2.6,4.8]	677	0.7	[-2.8,4.3]
Non-severe with doctor's diagnosis	988	1.5	[-2.5,5.4]	968	1.7	[-1.9,5.2]	632	-0.1	[-3.6,3.3]
Severe	286	18.4	[12.4,24.3]	280	13.5	[8.4,18.6]	191	-1.4	[-8.0,5.2]
Food allergy ever at age 9 yrs									
No	22,798	0.0	[0.0,0.0]	22,477	0.0	[0.0,0.0]	1,360	0.0	[0.0,0.0]
Yes	2,193	3.9	[1.2,6.5]	2,138	3.2	[0.8,5.5]	1,360	0.6	[-1.9,3.0]
**Non-eligibility to USS**		**OR**	**95% CI**		**OR**	**95% CI**		**OR**	**95% CI**
Food allergy in Years 7-9:									
No	1,683/22,669	1.00	[1.00,1.00]	1,656/22,352	1.00	[1.00,1.00]	54/104	1.00	[1.00,1.00]
Non-severe without doctor's diagnosis	54/919	0.78	[0.59,1.03]	52/892	0.82	[0.61,1.09]	22/52	0.74	[0.43,1.29]
Non-severe with doctor's diagnosis	74/1,047	0.95	[0.74,1.21]	72/1,026	0.97	[0.76,1.25]	25/48	1.08	[0.61,1.90]
Severe	10/194	0.68	[0.36,1.28]	7/189	0.53	[0.24,1.17]	5/8	1.69	[0.40,7.11]
Food allergy by Year 9:									
No	1,683/22,669	1.00	[1.00,1.00]	1,656/22,352	1.00	[1.00,1.00]	54/107	1.00	[1.00,1.00]

Non-severe without doctor's diagnosis	54/916	0.78	[0.59,1.03]	52/889	0.82	[0.61,1.09]	22/52	0.74	[0.43,1.29]
Non-severe with doctor's diagnosis	70/987	0.95	[0.74,1.22]	68/967	0.97	[0.75,1.26]	23/45	1.04	[0.58,1.88]
Severe	17/286	0.79	[0.48,1.29]	14/280	0.77	[0.44,1.35]	10/14	2.56	[0.80,8.20]
Food allergy ever at age 9 yrs	1,700/22,787	1.00	[1.00,1.00]	1,671/22,465	1.00	[1.00,1.00]	57/108	1.00	[1.00,1.00]
Yes	140/2,192	0.85	[0.71,1.01]	133/2,137	0.87	[0.72,1.05]	51/108	0.90	[0.61,1.31]

† Adjusted for gender, birth year, mother's and father's education, family income and parents' immigration status

‡ Adjusted for gender, birth year and family income

N=total number of individuals in the analysis; n=number of individuals with the outcome

Table E3. Results from the twin cohort: Associations between food allergy (both primary and secondary exposure measures) and the primary outcome Grade point sum and the secondary outcome non-eligibility to upper secondary school (USS); unadjusted, adjusted for measured confounders and additionally adjusted for confounders shared within twin pairs.

	Unadjusted			Adjusted†			Co-twin‡		
**Grade point sum in Year 9**	**N**	**β**	**95% CI**	**N**	**β**	**95% CI**	**N**	**β**	**95% CI**
Food allergy in Grades 7-9:									
No	22,684	0.0	[0.0,0.0]	22,365	0.0	[0.0,0.0]	1,321	0.0	[0.0,0.0]
Non-severe without doctor's diagnosis	919	2.4	[-1.7,6.5]	892	1.3	[-2.3,5.0]	677	1.0	[-2.6,4.6]
Non-severe with doctor's diagnosis	1,048	1.6	[-2.2,5.4]	1,027	1.5	[-1.9,4.9]	632	-0.1	[-3.5,3.2]
Severe	194	22.4	[15.4,29.4]	189	19.1	[13.2,25.1]	131	-0.4	[-8.5,7.7]
Food allergy by Grade 9:									
No	22,684	0.0	[0.0,0.0]	22,365	0.0	[0.0,0.0]	1,321	0.0	[0.0,0.0]
Non-severe without doctor's diagnosis	916	2.2	[-1.9,6.3]	889	1.2	[-2.5,4.9]	677	1.0	[-2.7,4.6]
Non-severe with doctor's diagnosis	988	1.4	[-2.6,5.3]	968	1.5	[-2.0,5.1]	632	-0.0	[-3.5,3.5]
Severe	286	18.5	[12.6,24.4]	280	13.8	[8.7,18.8]	191	-1.4	[-8.0,5.1]
Food allergy ever at age 9 years									
No	22,800	0.0	[0.0,0.0]	22,478	0.0	[0.0,0.0]	1,360	0.0	[0.0,0.0]
Yes	2,193	4.0	[1.3,6.6]	2,138	3.2	[0.8,5.6]	1,360	0.7	[-1.8,3.2]
**Non-eligibility to USS**	**n/N**	**OR**	**95% CI**	**n/N**	**OR**	**95% CI**	**n/N**	**OR**	**95% CI**
Food allergy in Grades 7-9:									
No	1,683/22,671	1.00	[1.00,1.00]	1,656/22,353	1.00	[1.00,1.00]	54/104	1.00	[1.00,1.00]
Non-severe without doctor's diagnosis	54/918	0.78	[0.59,1.02]	52/892	0.82	[0.61,1.09]	22/52	0.75	[0.44,1.29]
Non-severe with doctor's diagnosis	74/1,047	0.93	[0.73,1.18]	72/1,026	0.95	[0.74,1.22]	25/48	1.03	[0.59,1.81]
Severe	10/194	0.66	[0.35,1.25]	7/189	0.52	[0.24,1.14]	5/8	1.68	[0.40,7.09]
Food allergy by Grade 9:									
No	1,683/22,671	1.00	[1.00,1.00]	1,656/22,353	1.00	[1.00,1.00]	54/107	1.00	[1.00,1.00]

Non-severe without doctor's diagnosis	54/916	0.78	[0.59,1.03]	52/889	0.82	[0.61,1.09]	22/52	0.75	[0.44,1.29]
Non-severe with doctor's diagnosis	70/987	0.93	[0.73,1.19]	68/967	0.95	[0.73,1.23]	23/45	1.00	[0.56,1.78]
Severe	17/286	0.77	[0.47,1.25]	14/280	0.74	[0.42,1.30]	10/14	2.57	[0.80,8.23]
Food allergy ever at age 9 years									
No	1,700/22,787	1.00	[1.00,1.00]	1,671/22,466	1.00	[1.00,1.00]	57/108	1.00	[1.00,1.00]
Yes	140/2,192	0.83	[0.70,1.00]	133/2,137	0.86	[0.71,1.03]	51/108	0.88	[0.61,1.28]

† Adjusted for gender, birth year, mother's and father's education, family income and parents' immigration status

‡ Adjusted for gender

N=total number of individuals in the analysis; n=number of individuals with the outcome

Table E4. Results from the register-based cohort: Associations between food allergy (both primary and secondary exposure measures) and the secondary outcomes passing national tests (Year 3) or grades from national tests (Years 6 and 9); unadjusted, adjusted for measured confounders and additionally adjusted for confounders shared between siblings.

	**Subject**		**Crude**			**Adjusted**[†]			**Sibling**[‡]	
**Year 3**		**n/N**	**OR**	**95% CI**	**n/N**	**OR**	**95% CI**	**n/N**	**OR**	**95% CI**
**Food allergy in Years 1-3:**										
No	Swedish	332,391/432,508	1.00	[1.00,1.00]	325,569/422,298	1.00	[1.00,1.00]	460/894	1.00	[1.00,1.00]
Non-severe		5,341/6,990	0.98	[0.92,1.03]	5,233/6,833	0.99	[0.93,1.05]	306/630	0.94	[0.80,1.11]
Severe		1,901/2,355	1.26	[1.14,1.40]	1,869/2,308	1.19	[1.07,1.33]	118/218	1.27	[0.95,1.69]
No	Mathematics	306,487/432,877	1.00	[1.00,1.00]	300,384/422,647	1.00	[1.00,1.00]	532/1,048	1.00	[1.00,1.00]
Non-severe		4,959/7,011	1.00	[0.95,1.05]	4,870/6,856	1.01	[0.95,1.06]	370/747	0.99	[0.86,1.15]
Severe		1,782/2,352	1.29	[1.17,1.42]	1,754/2,307	1.16	[1.05,1.29]	135/262	1.03	[0.81,1.32]
Food allergy severity ever by Year 3:										
No	Swedish	332,391/432,508	1.00	[1.00,1.00]	325,569/422,298	1.00	[1.00,1.00]	460/894	1.00	[1.00,1.00]
Non-severe		4,917/6,449	0.97	[0.91,1.02]	4,817/6,305	0.98	[0.92,1.04]	291/592	0.95	[0.81,1.13]
Severe		2,325/2,896	1.23	[1.12,1.34]	2,285/2,836	1.18	[1.07,1.30]	137/263	1.17	[0.90,1.53]
No	Mathematics	306,487/432,877	1.00	[1.00,1.00]	300,384/422,647	1.00	[1.00,1.00]	532/1,048	1.00	[1.00,1.00]
Non-severe		4,578/6,468	1.00	[0.95,1.05]	4,494/6,325	1.01	[0.96,1.07]	348/697	1.00	[0.86,1.17]
Severe		2,163/2,895	1.22	[1.12,1.33]	2,130/2,838	1.11	[1.02,1.22]	162/321	1.00	[0.80,1.25]
**Food allergy ever by Year 3:**										
No	Swedish	332,391/432,508	1.00	[1.00,1.00]	325,569/422,298	1.00	[1.00,1.00]	460/894	1.00	[1.00,1.00]
Yes		7,242/9,345	1.04	[0.99,1.09]	7,102/9,141	1.03	[0.98,1.09]	413/825	1.02	[0.88,1.17]
No	Mathematics	306,487/432,877	1.00	[1.00,1.00]	300,384/422,647	1.00	[1.00,1.00]	532/1,048	1.00	[1.00,1.00]
Yes		6,741/9,363	1.06	[1.01,1.11]	6,624/9,163	1.04	[0.99,1.09]	485/968	1.00	[0.88,1.14]
**Year 6**		**N**	**β**	**95% CI**	**N**	**β**	**95% CI**	**N**	**β**	**95% CI**

**Food allergy in Years 4-6:**										
No	Swedish	422,963	0.0	[0.0,0.0]	413,024	0.0	[0.0,0.0]	2,610	0.0	[0.0,0.0]
Non-severe		8,197	0.0	[-0.1,0.1]	8,010	0.1	[0.0,0.2]	1,845	-0.0	[-0.2,0.2]
Severe		2,939	0.5	[0.4,0.6]	2,882	0.3	[0.2,0.5]	723	0.1	[-0.1,0.4]
No	Mathematics	424,057	0.0	[0.0,0.0]	414,090	0.0	[0.0,0.0]	2,660	0.0	[0.0,0.0]
Non-severe		8,219	0.1	[-0.0,0.2]	8,032	0.1	[-0.0,0.2]	1,893	0.0	[-0.2,0.2]
Severe		2,956	0.8	[0.6,0.9]	2,898	0.4	[0.2,0.5]	717	0.2	[-0.1,0.6]
No	English	423,652	0.0	[0.0,0.0]	413,709	0.0	[0.0,0.0]	2,526	0.0	[0.0,0.0]
Non-severe		8,208	0.1	[-0.0,0.2]	8,023	-0.0	[-0.1,0.1]	1,790	-0.2	[-0.4,-0.0]
Severe		2,960	0.7	[0.6,0.9]	2,899	0.4	[0.2,0.5]	685	0.2	[-0.1,0.4]
**Food allergy severity ever by Year 6:**										
No	Swedish	422,963	0.0	[0.0,0.0]	413,024	0.0	[0.0,0.0]	2,610	0.0	[0.0,0.0]
Non-severe		7,320	-0.0	[-0.1,0.1]	7,152	0.1	[-0.0,0.1]	1,673	0.0	[-0.2,0.2]
Severe		3,816	0.4	[0.3,0.6]	3,740	0.3	[0.2,0.4]	929	0.1	[-0.2,0.3]
No	Mathematics	424,057	0.0	[0.0,0.0]	414,090	0.0	[0.0,0.0]	2,660	0.0	[0.0,0.0]
Non-severe		7,343	0.0	[-0.1,0.2]	7,174	0.1	[-0.0,0.2]	1,707	-0.0	[-0.2,0.2]
Severe		3,832	0.7	[0.5,0.8]	3,756	0.3	[0.2,0.4]	930	0.3	[0.0,0.6]
No	English	423,652	0.0	[0.0,0.0]	413,709	0.0	[0.0,0.0]	2,526	0.0	[0.0,0.0]
Non-severe		7,327	0.0	[-0.1,0.1]	7,161	-0.0	[-0.1,0.1]	1,613	-0.3	[-0.4,-0.1]
Severe		3,841	0.6	[0.5,0.8]	3,761	0.3	[0.2,0.4]	890	0.1	[-0.1,0.4]
**Food allergy ever by Year 6:**										
No	Swedish	422,963	0.0	[0.0,0.0]	413,024	0.0	[0.0,0.0]	2,610	0.0	[0.0,0.0]
Yes		11,136	0.1	[0.1,0.2]	10,892	0.1	[0.1,0.2]	2,484	0.0	[-0.1,0.2]
No	Mathematics	424,057	0.0	[0.0,0.0]	414,090	0.0	[0.0,0.0]	2,660	0.0	[0.0,0.0]

Yes		11,175	0.3	[0.2,0.4]	10,930	0.1	[0.1,0.2]	2,524	0.1	[-0.1,0.3]
No	English	423,652	0.0	[0.0,0.0]	413,709	0.0	[0.0,0.0]	2,526	0.0	[0.0,0.0]
Yes		11,168	0.2	[0.2,0.3]	10,922	0.1	[0.0,0.2]	2,391	-0.1	[-0.3,0.0]
**Year 9**		**N**	**β**	**95% CI**	**N**	**β**	**95% CI**	**N**	**β**	**95% CI**
**Food allergy in Years 7-9:**										
No	Swedish	215,387	0.0	[0.0,0.0]	210,948	0.0	[0.0,0.0]	521	0.0	[0.0,0.0]
Non-severe		4,184	0.1	[-0.1,0.2]	4,094	0.1	[-0.0,0.2]	376	-0.2	[-0.6,0.2]
Severe		1,713	0.6	[0.4,0.8]	1,686	0.3	[0.1,0.5]	168	0.0	[-0.5,0.6]
No	Mathematics	176,992	0.0	[0.0,0.0]	173,339	0.0	[0.0,0.0]	478	0.0	[0.0,0.0]
Non-severe		3,401	0.1	[-0.1,0.3]	3,325	0.2	[-0.0,0.3]	347	0.1	[-0.4,0.7]
Severe		1,414	1.0	[0.7,1.2]	1,396	0.5	[0.2,0.7]	151	-0.0	[-0.8,0.7]
No	English	225,132	0.0	[0.0,0.0]	220,532	0.0	[0.0,0.0]	528	0.0	[0.0,0.0]
Non-severe		4,343	0.2	[0.0,0.3]	4,250	0.1	[0.0,0.2]	370	-0.4	[-0.7,-0.1]
Severe		1,821	0.7	[0.5,0.8]	1,792	0.4	[0.2,0.5]	179	-0.0	[-0.5,0.4]
**Food allergy severity ever by Year 9:**										
No	Swedish	215,387	0.0	[0.0,0.0]	210,948	0.0	[0.0,0.0]	521	0.0	[0.0,0.0]
Non-severe		3,595	0.0	[-0.1,0.2]	3,518	0.1	[-0.0,0.2]	335	-0.1	[-0.5,0.3]
Severe		2,302	0.5	[0.4,0.7]	2,262	0.3	[0.2,0.5]	231	-0.1	[-0.6,0.4]
No	Mathematics	176,992	0.0	[0.0,0.0]	173,339	0.0	[0.0,0.0]	478	0.0	[0.0,0.0]
Non-severe		2,908	0.1	[-0.1,0.3]	2,841	0.2	[-0.0,0.3]	302	0.2	[-0.4,0.7]
Severe		1,907	0.8	[0.5,1.0]	1,880	0.4	[0.2,0.6]	204	-0.0	[-0.6,0.6]
No	English	225,132	0.0	[0.0,0.0]	220,532	0.0	[0.0,0.0]	528	0.0	[0.0,0.0]
Non-severe		3,730	0.1	[-0.1,0.2]	3,650	0.1	[-0.1,0.2]	334	-0.4	[-0.8,-0.1]
Severe		2,434	0.7	[0.6,0.8]	2,392	0.4	[0.3,0.6]	235	-0.0	[-0.4,0.3]

**Food allergy ever by Year 9:**										
No	Swedish	215,387	0.0	[0.0,0.0]	210,948	0.0	[0.0,0.0]	521	0.0	[0.0,0.0]
Yes		5,897	0.2	[0.1,0.3]	5,780	0.2	[0.1,0.3]	513	-0.1	[-0.5,0.2]
No	Mathematics	176,992	0.0	[0.0,0.0]	173,339	0.0	[0.0,0.0]	478	0.0	[0.0,0.0]
Yes		4,815	0.4	[0.2,0.5]	4,721	0.2	[0.1,0.4]	474	0.1	[-0.4,0.5]
No	English	225,132	0.0	[0.0,0.0]	220,532	0.0	[0.0,0.0]	528	0.0	[0.0,0.0]
Yes		6,164	0.3	[0.2,0.4]	6,042	0.2	[0.1,0.3]	519	-0.3	[-0.5,-0.0]

† Adjusted for gender, birth year, mother's and father's education, family income and parents' immigration status

‡ Adjusted for gender

N=total number of individuals in the analysis; n=number of individuals with the outcome

Table E5. Results from twin cohort: Associations between food allergy (both primary and secondary exposure measures) and the secondary outcomes passing national tests (Year 3) or grades from national tests (Years 6 and 9); unadjusted, adjusted for measured confounders and additionally adjusted for confounders shared within twin pairs.

		Crude			Adjusted†			Co-twin‡		
**Year 3**		**n/N**	**OR**	**95% CI**	**n/N**	**OR**	**95% CI**	**n/N**	**OR**	**95% CI**
	**Food allergy in Years 1-3:**									
Swedish	No	11,737/15,037	1.00	[1.00,1.00]	11,517/14,744	1.00	[1.00,1.00]	107/215	1.00	[1.00,1.00]
	Non-severe without doctor's diagnosis	475/596	1.10	[0.90,1.35]	454/574	0.98	[0.79,1.22]	50/107	0.77	[0.51,1.15]
	Non-severe with doctor's diagnosis	640/799	1.13	[0.95,1.35]	620/772	1.12	[0.93,1.36]	61/119	0.97	[0.66,1.42]
	Severe	56/63	2.25	[1.02,4.94]	55/62	2.26	[1.03,4.98]	8/11	1.66	[0.42,6.53]
Mathematics	No	10,670/15,053	1.00	[1.00,1.00]	10,464/14,761	1.00	[1.00,1.00]	134/250	1.00	[1.00,1.00]
	Non-severe without doctor's diagnosis	419/599	0.96	[0.80,1.14]	400/577	0.88	[0.73,1.06]	59/123	0.92	[0.64,1.31]
	Non-severe with doctor's diagnosis	565/795	1.01	[0.86,1.18]	548/768	0.97	[0.83,1.15]	58/127	0.83	[0.58,1.18]
	Severe	42/63	0.82	[0.49,1.39]	41/62	0.79	[0.47,1.35]	9/20	0.84	[0.34,2.03]
	**Food allergy by Year 3:**									
Swedish	No	11,737/15,037	1.00	[1.00,1.00]	11,523/14,753	1.00	[1.00,1.00]	107/215	1.00	[1.00,1.00]
	Non-severe without doctor's diagnosis	473/594	1.10	[0.90,1.35]	452/572	0.98	[0.79,1.21]	49/106	0.76	[0.50,1.14]
	Non-severe with doctor's diagnosis	623/778	1.13	[0.94,1.35]	603/752	1.12	[0.92,1.35]	62/119	1.01	[0.69,1.48]
	Severe	77/88	1.97	[1.05,3.71]	76/86	2.11	[1.08,4.12]	8/12	1.06	[0.31,3.69]
Mathematics	No	10,670/15,053	1.00	[1.00,1.00]	10,464/14,761	1.00	[1.00,1.00]	134/250	1.00	[1.00,1.00]

	Non-severe without doctor's diagnosis	418/597	0.96	[0.80,1.15]	399/575	0.88	[0.73,1.06]	59/123	0.92	[0.64,1.31]
	Non-severe with doctor's diagnosis	551/776	1.01	[0.86,1.18]	534/750	0.97	[0.82,1.14]	58/125	0.85	[0.60,1.21]
	Severe	59/86	0.90	[0.57,1.42]	58/84	0.86	[0.54,1.38]	8/20	0.69	[0.28,1.70]
	**Food allergy ever at age 9 yrs**									
Swedish	No	11,775/15,083	1.00	[1.00,1.00]	11,554/14,788	1.00	[1.00,1.00]	108/219	1.00	[1.00,1.00]
	Yes	1,192/1,482	1.15	[1.01,1.32]	1,150/1,431	1.10	[0.95,1.27]	111/219	0.90	[0.68,1.19]
Mathematics	No	10,700/15,099	1.00	[1.00,1.00]	10,493/14,805	1.00	[1.00,1.00]	136/258	1.00	[1.00,1.00]
	Yes	1,047/1,483	0.99	[0.88,1.11]	1,010/1,432	0.93	[0.83,1.06]	122/258	0.90	[0.71,1.15]

**Year 6**		**N**	**β**	**95% CI**	**N**	**β**	**95% CI**	**N**	**β**	**95% CI**
	**Food allergy in Years 4-6:**									
Swedish	No	10,703	0.0	[0.0,0.0]	10,519	0.0	[0.0,0.0]	399	0.0	[0.0,0.0]
	Non-severe without doctor's diagnosis	410	0.1	[-0.3,0.5]	391	-0.1	[-0.5,0.3]	193	-0.3	[-0.7,0.1]
	Non-severe with doctor's diagnosis	537	0.2	[-0.1,0.6]	523	0.1	[-0.2,0.5]	212	0.0	[-0.3,0.4]
	Severe	63	1.2	[0.5,2.0]	62	1.1	[0.4,1.8]	32	-0.5	[-1.5,0.4]
Mathematics	No	10,726	0.0	[0.0,0.0]	10,540	0.0	[0.0,0.0]	421	0.0	[0.0,0.0]
	Non-severe without doctor's diagnosis	406	-0.3	[-0.8,0.2]	387	-0.4	[-0.9,0.1]	203	-0.5	[-1.0,-0.1]
	Non-severe with doctor's diagnosis	547	0.2	[-0.2,0.7]	533	0.1	[-0.3,0.6]	218	-0.1	[-0.7,0.4]
	Severe	64	0.8	[-0.3,1.9]	63	0.7	[-0.4,1.7]	38	-0.1	[-1.6,1.5]

English	No	10,680	0.0	[0.0,0.0]	10,495	0.0	[0.0,0.0]	405	0.0	[0.0,0.0]
	Non-severe without doctor's diagnosis	403	0.1	[-0.4,0.6]	384	0.1	[-0.4,0.5]	198	-0.1	[-0.6,0.4]
	Non-severe with doctor's diagnosis	544	0.4	[-0.0,0.7]	528	0.3	[-0.0,0.7]	217	0.5	[-0.0,0.9]
	Severe	61	1.2	[0.1,2.3]	60	1.2	[0.1,2.2]	30	-0.1	[-1.3,1.0]
	**Food allergy by Year 6:**									
Swedish	No	10,703	0.0	[0.0,0.0]	10,519	0.0	[0.0,0.0]	404	0.0	[0.0,0.0]
	Non-severe without doctor's diagnosis	407	0.1	[-0.3,0.5]	388	-0.1	[-0.5,0.3]	192	-0.3	[-0.6,0.1]
	Non-severe with doctor's diagnosis	508	0.2	[-0.1,0.6]	495	0.2	[-0.1,0.5]	203	0.0	[-0.3,0.4]
	Severe	102	0.8	[0.1,1.4]	100	0.6	[0.0,1.2]	51	-0.6	[-1.4,0.1]
Mathematics	No	10,726	0.0	[0.0,0.0]	10,540	0.0	[0.0,0.0]	424	0.0	[0.0,0.0]
	Non-severe without doctor's diagnosis	403	-0.3	[-0.8,0.2]	384	-0.4	[-0.9,0.1]	203	-0.5	[-1.0,-0.1]
	Non-severe with doctor's diagnosis	518	0.2	[-0.2,0.7]	505	0.2	[-0.2,0.6]	206	-0.0	[-0.6,0.5]
	Severe	103	0.6	[-0.3,1.6]	101	0.4	[-0.6,1.3]	51	-0.9	[-2.0,0.3]
English	No	10,680	0.0	[0.0,0.0]	10,495	0.0	[0.0,0.0]	407	0.0	[0.0,0.0]
	Non-severe without doctor's diagnosis	400	0.1	[-0.4,0.6]	381	0.1	[-0.4,0.5]	196	-0.1	[-0.5,0.4]
	Non-severe with doctor's diagnosis	515	0.3	[-0.1,0.7]	500	0.3	[-0.1,0.7]	207	0.4	[-0.0,0.9]
	Severe	100	1.0	[0.2,1.8]	98	0.8	[0.1,1.6]	42	-0.2	[-1.1,0.7]

		N	β	95% CI	N	β	95% CI	N	β	95% CI
	**Food allergy ever at age 9 yrs**									
Swedish	No	10,768	0.0	[0.0,0.0]	10,581	0.0	[0.0,0.0]	418	0.0	[0.0,0.0]
	Yes	1,027	0.3	[0.0,0.5]	993	0.1	[-0.1,0.4]	418	-0.1	[-0.4,0.1]
Mathematics	No	10,789	0.0	[0.0,0.0]	10,600	0.0	[0.0,0.0]	433	0.0	[0.0,0.0]
	Yes	1,034	0.1	[-0.2,0.4]	1,000	-0.0	[-0.3,0.3]	433	-0.3	[-0.6,0.0]
English	No	10,743	0.0	[0.0,0.0]	10,555	0.0	[0.0,0.0]	420	0.0	[0.0,0.0]
	Yes	1,025	0.3	[0.0,0.6]	989	0.3	[-0.0,0.6]	420	0.2	[-0.2,0.5]
**Year 9**		**N**	**β**	**95% CI**	**N**	**β**	**95% CI**	**N**	**β**	**95% CI**
	**Food allergy in Years 7-9:**									
Swedish	No	18,747	0.0	[0.0,0.0]	18,507	0.0	[0.0,0.0]	607	0.0	[0.0,0.0]
	Non-severe without doctor's diagnosis	757	0.3	[0.0,0.6]	743	0.2	[-0.0,0.5]	327	0.1	[-0.2,0.4]
	Non-severe with doctor's diagnosis	845	0.0	[-0.2,0.3]	832	0.1	[-0.2,0.3]	306	-0.2	[-0.5,0.1]
	Severe	158	0.9	[0.3,1.5]	154	0.9	[0.3,1.4]	68	-0.7	[-1.5,0.0]
Mathematics	No	17,752	0.0	[0.0,0.0]	17,526	0.0	[0.0,0.0]	626	0.0	[0.0,0.0]
	Non-severe without doctor's diagnosis	720	0.3	[-0.1,0.7]	704	0.2	[-0.1,0.6]	330	-0.1	[-0.6,0.3]
	Non-severe with doctor's diagnosis	778	0.6	[0.2,0.9]	767	0.4	[0.1,0.7]	305	-0.3	[-0.8,0.1]
	Severe	147	0.9	[-0.1,1.8]	144	0.5	[-0.4,1.4]	61	-0.9	[-2.0,0.2]
English	No	18,888	0.0	[0.0,0.0]	18,640	0.0	[0.0,0.0]	570	0.0	[0.0,0.0]
	Non-severe without doctor's diagnosis	762	0.3	[0.1,0.6]	745	0.4	[0.1,0.6]	297	-0.0	[-0.3,0.2]

	Non-severe with doctor's diagnosis	844	0.1	[0.0,0.4]	813	0.1	[-0.1,0.4]	289	-0.1	[-0.5,0.2]
	Severe	160	1.0	[0.5,1.6]	155	1.0	[0.5,1.5]	50	-0.5	[-1.1,0.1]
	**Food allergy by Year 9:**									
Swedish	No	18,747	0.0	[0.0,0.0]	18,507	0.0	[0.0,0.0]	617	0.0	[0.0,0.0]
	Non-severe without doctor's diagnosis	755	0.3	[0.0,0.6]	741	0.2	[-0.0,0.5]	325	0.1	[-0.2,0.4]
	Non-severe with doctor's diagnosis	797	0.0	[-0.2,0.3]	785	0.1	[-0.1,0.4]	284	-0.2	[-0.5,0.1]
	Severe	226	0.8	[0.2,1.3]	221	0.6	[0.1,1.1]	98	-0.5	[-1.1,0.2]
Mathematics	No	17,752	0.0	[0.0,0.0]	17,526	0.0	[0.0,0.0]	634	0.0	[0.0,0.0]
	Non-severe without doctor's diagnosis	718	0.3	[-0.1,0.7]	702	0.2	[-0.1,0.6]	329	-0.2	[-0.6,0.3]
	Non-severe with doctor's diagnosis	732	0.6	[0.2,0.9]	722	0.4	[0.1,0.8]	287	-0.3	[-0.8,0.1]
	Severe	215	0.9	[0.1,1.6]	211	0.3	[-0.4,1.0]	86	-0.6	[-1.4,0.3]
English	No	18,888	0.0	[0.0,0.0]	18,640	0.0	[0.0,0.0]	578	0.0	[0.0,0.0]
	Non-severe without doctor's diagnosis	759	0.3	[0.0,0.6]	742	0.4	[0.1,0.6]	295	-0.0	[-0.3,0.2]
	Non-severe with doctor's diagnosis	796	0.1	[-0.2,0.4]	784	0.1	[-0.2,0.4]	273	-0.2	[-0.5,0.2]
	Severe	233	1.2	[0.7,1.6]	227	0.9	[0.5,1.3]	76	-0.3	[-0.8,0.2]
	**Food allergy ever at age 9 yrs**									
Swedish	No	18,820	0.0	[0.0,0.0]	18,580	0.0	[0.0,0.0]	629	0.0	[0.0,0.0]
	Yes	1,786	0.3	[0.1,0.4]	1,753	0.2	[0.1,0.4]	629	-0.1	[-0.3,0.1]

Mathematics	No	17,825	0.0	[0.0,0.0]	17,599	0.0	[0.0,0.0]	649	0.0	[0.0,0.0]
	Yes	1,672	0.5	[0.2,0.8]	1,641	0.3	[0.1,0.6]	649	-0.2	[-0.6,0.1]
English	No	18,966	0.0	[0.0,0.0]	18,718	0.0	[0.0,0.0]	589	0.0	[0.0,0.0]
	Yes	1,794	0.3	[0.1,0.5]	1,757	0.3	[0.1,0.5]	589	-0.1	[-0.3,0.1]

† Adjusted for gender, birth year, mother's and father's education, family income and parents' immigration status

‡ Adjusted for gender

N=total number of individuals in the analysis; n=number of individuals with the outcome

Table E6. Results from the register-based cohort: Associations between food allergy severity and school performance measures by gender, using sibling controls with fixed effects linear regression or conditional logistic regression in register-based cohort.

	Boys			Girls		
**Grade point sum in Year 9**	**N**	**β**	**95% CI**	**N**	**β**	**95% CI**
No	2,093	0.0		2,121	0.0	
Non-severe	1,619	-0.4	[-3.2,2.3]	1,382	0.4	[-2.8,3.6]
Severe	668	1.3	[-2.8,5.5]	528	2.5	[-2.2,7.1]
**Non-eligibility to USS**	**n/N**	**OR**	**95% CI**	**n/N**	**OR**	**95% CI**
No	107/199	1.00		85/201	1.00	
Non-severe	87/152	1.31	[0.94,1.83]	56/132	0.84	[0.59,1.19]
Severe	32/55	1.21	[0.69,2.10]	19/42	1.02	[0.55,1.90]
**Passing national test in Year 3 - Swedish**	**n/N**	**OR**	**95% CI**	**n/N**	**OR**	**95% CI**
No	209/486	1.00		251/408	1.00	
Non-severe	146/362	0.96	[0.78,1.19]	160/268	0.92	[0.72,1.17]
Severe	55/130	1.09	[0.76,1.57]*	63/88	1.74	[1.08,2.82]
**Passing national test in Year 3 - Mathematics**	**n/N**	**OR**	**95% CI**	**n/N**	**OR**	**95% CI**
No	256/527	1.00		276/521	1.00	
Non-severe	222/426	1.05	[0.87,1.27]	148/321	0.92	[0.74,1.14]
Severe	71/146	0.93	[0.67,1.29]	64/116	1.27	[0.87,1.86]
**National test score in Year 6 - Swedish**	**N**	**β**	**95% CI**	**N**	**β**	**95% CI**
No	1,296	0.0		1,314	0.0	
Non-severe	994	0.0	[-0.2,0.3]	851	-0.1	[-0.3,0.2]
Severe	412	0.2	[-0.1,0.5]	311	0.0	[-0.3,0.4]
**National test score in Year 6 - Mathematics**	**N**	**β**	**95% CI**	**N**	**β**	**95% CI**
No	1,294	0.0		1,366	0.0	
Non-severe	1,018	0.1	[-0.2,0.3]	875	-0.0	[-0.3,0.2]
Severe	399	0.4	[-0.0,0.8]	318	0.1	[-0.4,0.5]
**National test score in Year 6 - English**	**N**	**β**	**95% CI**	**N**	**β**	**95% CI**
No	1,265	0.0		1,261	0.0	
Non-severe	962	-0.1	[-0.4,0.1]	828	-0.3	[-0.6,-0.0]

Severe	374	0.2	[-0.2,0.5]	311	0.2	[-0.3,0.6]
**National test score in Year 9 - Swedish**	**N**	**β**	**95% CI**	**N**	**β**	**95% CI**
No	256	0.0		265	0.0	
Non-severe	217	-0.1	[-0.6,0.4]	159	-0.3	[-0.9,0.2]
Severe	93	0.0	[-0.8,0.8]	75	0.1	[-0.7,0.9]
**National test score in Year 9 - Mathematics**	**N**	**β**	**95% CI**	**N**	**β**	**95% CI**
No	236	0.0		242	0.0	
Non-severe	204	0.6	[-0.1,1.3]*	143	-0.5	[-1.2,0.2]
Severe	82	0.2	[-0.7,1.2]	69	-0.4	[-1.5,0.7]
**National test score in Year 9 - English**	**N**	**β**	**95% CI**	**N**	**β**	**95% CI**
No	262	0.0		266	0.0	
Non-severe	218	-0.4	[-0.7,-0.0]	152	-0.4	[-0.9,0.0]
Severe	103	0.1	[-0.5,0.6]	76	-0.1	[-0.8,0.6]

* p-value<0.05 for difference in association between girls and boys

N=total number of individuals from exposure and outcome discordant sibling groups

n=number of individuals from exposure and outcome discordant sibling groups with the outcome

Table E7. Results from the register-based cohort: Associations between food allergy severity and school performance measures by parental education, using sibling controls with fixed effects linear regression or conditional logistic regression in register-based cohort.

	Lower secondary school			Upper secondary school			University		
**Grade point sum in Year 9**	**N**	**β**	**95% CI**	**N**	**β**	**95% CI**	**N**	**β**	**95% CI**
Food allergy severity: No	84	0.0		1,405	0.0		2,723	0.0	
Non-severe	68	6.6	[-9.7,23.0]	1,042	-2.8	[-6.8,1.1]	1,889	1.3	[-1.2,3.7]
Severe	13	-42.4	[-77.0,-7.9]†	341	2.3	[-4.6,9.2]	842	2.1	[-1.3,5.5]
**Non-eligibility to USS**	**n/N**	**OR**	**95% CI**	**n/N**	**OR**	**95% CI**	**n/N**	**OR**	**95% CI**
Food allergy severity: No	5/20	1.00		116/241	1.00		70/138	1.00	
Non-severe	11/18	1.73	[0.64,4.64]	88/175	1.05	[0.78,1.42]	44/90	0.94	[0.62,1.42]
Severe	3/4	2.86	[0.29,28.53]	28/52	1.21	[0.70,2.11]	20/41	0.98	[0.52,1.83]
**Passing national test in Year 3 - Swedish**	**n/N**	**OR**	**95% CI**	**n/N**	**OR**	**95% CI**	**n/N**	**OR**	**95% CI**
Food allergy severity: No	18/29	1.00		186/370	1.00		256/494	1.00	
Non-severe	9/19	0.85	[0.33,2.20]	131/268	0.98	[0.76,1.25]	165/342	0.92	[0.74,1.15]
Severe	1/4	0.24	[0.02,2.84]	43/76	1.36	[0.85,2.18]	74/138	1.27	[0.89,1.80]
**Passing national test in Year 3 - Mathematics**	**n/N**	**OR**	**95% CI**	**n/N**	**OR**	**95% CI**	**n/N**	**OR**	**95% CI**
Food allergy severity: No	15/30	1.00		210/403	1.00		306/613	1.00	
Non-severe	12/20	1.57	[0.62,4.00]	149/308	0.93	[0.74,1.17]	208/417	1.01	[0.83,1.23]
Severe	1/5	0.29	[0.03,2.68]	44/81	1.18	[0.75,1.84]	90/176	1.00	[0.74,1.36]
**National test score in Year 6 - Swedish**	**N**	**β**	**95% CI**	**N**	**β**	**95% CI**	**N**	**β**	**95% CI**
Food allergy severity: No	58	0.0		852	0.0		1,700	0.0	
Non-severe	46	-0.3	[-1.8,1.2]	642	-0.0	[-0.3,0.3]	1,157	-0.0	[-0.2,0.2]
Severe	9	-0.6	[-4.5,3.3]	202	0.3	[-0.2,0.8]	512	0.1	[-0.2,0.4]
**National test score in Year 6 - Mathematics**	**N**	**β**	**95% CI**	**N**	**β**	**95% CI**	**N**	**β**	**95% CI**
Food allergy severity: No	51	0.0		865	0.0		1,742	0.0	
Non-severe	36	-0.2	[-1.7,1.4]	643	-0.2	[-0.5,0.2]	1,212	0.1	[-0.1,0.4]
Severe	9	-0.2	[-3.3,2.9]	199	0.4	[-0.2,1.1]	509	0.2	[-0.1,0.5]
**National test score in Year 6 - English**	**N**	**β**	**95% CI**	**N**	**β**	**95% CI**	**N**	**β**	**95% CI**
Food allergy severity: No	55	0.0		855	0.0		1,615	0.0	
Non-severe	41	0.2	[-1.2,1.6]	626	-0.5	[-0.9,-0.2]‡	1,122	-0.1	[-0.3,0.1]

Severe	11	0.6	[-2.6,3.7]	200	0.2	[-0.4,0.7]	474	0.2	[-0.2,0.5]
**National test score in Year 9 - Swedish**	**N**	**β**	**95% CI**	**N**	**β**	**95% CI**	**N**	**β**	**95% CI**
Food allergy severity: No	12	0.0		178	0.0		331	0.0	
Non-severe	11	0.0	[-3.4,3.5]	130	-0.4	[-1.1,0.3]	235	-0.1	[-0.5,0.4]
Severe	1	2.5	[0.7,4.2]§	55	-0.4	[-1.7,0.9]	112	0.2	[-0.4,0.8]
**National test score in Year 9 - Mathematics**	**N**	**β**	**95% CI**	**N**	**β**	**95% CI**	**N**	**β**	**95% CI**
Food allergy severity: No	10	0.0		144	0.0		324	0.0	
Non-severe	7	-1.7	[-6.0,2.7]	106	-0.0	[-1.0,1.0]	234	0.3	[-0.3,0.9]
Severe	4	0.2	[-5.0,5.5]	43	-0.6	[-2.2,1.1]	104	0.2	[-0.6,1.0]
**National test score in Year 9 - English**	**N**	**β**	**95% CI**	**N**	**β**	**95% CI**	**N**	**β**	**95% CI**
Food allergy severity: No	13	0.0		193	0.0		322	0.0	
Non-severe	11	-0.7	[-3.1,1.8]	143	-0.3	[-0.9,0.3]	213	-0.4	[-0.8,-0.1]
Severe	3	-2.3	[-4.2,-0.3]	56	-0.2	[-1.1,0.7]	120	0.2	[-0.4,0.7]

† p-value for Lower secondary school vs Upper secondary school = 0.013; Lower secondary school vs University =0.009

‡ p-value for Upper secondary school vs University = 0.036

§ p-value for Lower secondary school vs Upper secondary school = 0.016; Lower secondary school vs University =0.009

N=total number of individuals from exposure and outcome discordant sibling groups

Table E8. Results from register-based cohort: Associations between number of foods and type of food that causes allergic reaction and the primary outcome Grade point sum in Year 9; unadjusted, adjusted for measured confounders and additionally adjusted for confounders shared between siblings.

		Crude			Adjusted[†]			Sibling[‡]	
	**N**=450,437	**β**	**95% CI**	**N**=439,703	**β**	**95% CI**		**β**	**95% CI**
Number of foods (0-5)		2.3	[1.6,3.0]		1.3	[0.7,1.9]	8,472	0.5	[-0.7,1.6]
**Type of food:**	**n**			**n**			**n/N**		
Cow's milk	4,728	-0.9	[-2.8,1.0]	4,626	-1.5	[-3.2,0.2]	1,451/2,965	-2.0	[-5.0,1.0]
Egg	3,529	7.2	[5.2,9.2]	3,440	4.4	[2.6,6.2]	1,048/2,148	2.7	[-0.6,6.1]
Nuts	6,263	6.9	[5.4,8.5]	6,125	4.5	[3.1,5.9]	2,073/ 4,252	2.5	[-0.0,5.1]
Fish/crustacean	855	0.7	[-3.7,5.1]	831	-0.6	[-4.6,3.4]	299/622	-4.2	[-11.2,2.8]
Other foods	2,978	4.1	[1.8,6.4]	2,904	2.2	[0.1,4.4]	971/1,998	0.6	[-3.0,4.1]

[†] Adjusted for gender, birth year, mother's and father's education, family income and parents' immigration status

[‡] Adjusted for gender, birth year and family income

N=total number of individuals in the analysis; n=number of individuals with the exposure in the analysis
